# Hyperuricemia-related erectile dysfunction: mechanistic links and a translational framework for integrated traditional Chinese and western medicine

**DOI:** 10.3389/fendo.2026.1911364

**Published:** 2026-10-22

**Authors:** Shuo Huang, Yini Fang, Maojun Jiang, Yulin Zong, Yunzhi Zhang, Hanbo Ma, Linghui Zhu, Xi Zhang, Yunlong Yao, Yaxin Cao, Can Yang, Jianfeng Zeng, Lingru Li, Wenlong Sun, Yanfei Zheng

**Affiliations:** 1 School of Traditional Chinese Medicine, Beijing University of Chinese Medicine, Beijing, China; 2 Wangqi Academy, National Institute of TCM Constitution and Preventive Medicine, Beijing University of Chinese Medicine, Beijing, China; 3 Innovation Institute of Integrated Traditional Chinese and Western Medicine, Shandong First Medical University & Shandong Academy of Medical Sciences, Jinan, Shandong, China; 4 Zhongshan Hospital of Traditional Chinese Medicine Affiliated to Guangzhou University of Chinese Medicine, Zhongshan, Guangdong, China; 5 School of Life Sciences and Medicine, Shandong University of Technology, Zibo, Shandong, China

**Keywords:** clinical translation, erectile dysfunction, hyperuricemia, integration of traditional Chinese and western medicine, mechanism integration

## Abstract

Hyperuricemia-associated erectile dysfunction (HUAED) is an emerging, heterogeneous comorbidity linked to metabolic dysregulation, chronic inflammation, oxidative stress, and neurovascular dysfunction. Current evidence supports an epidemiological association between hyperuricemia (HUA) and erectile dysfunction (ED), but does not establish a uniform causal relationship. Mechanistic studies suggest that HUA may impair neural initiation and endothelial maintenance of erection, reduce nitric oxide (NO) bioavailability, disturb soluble guanylate cyclase (sGC)/cyclic guanosine monophosphate (cGMP) signaling, activate inflammatory pathways, and alter cavernosal smooth-muscle tone. Direct human evidence for HUA-driven penile fibrosis remains limited. Western management currently treats hyperuricemia and ED according to their established indications, whereas traditional Chinese medicine (TCM) has been proposed as a complementary, multi-target research domain. We therefore synthesize epidemiological, clinical, and preclinical evidence and propose a hypothesis-generating prevention-regulation-repair framework organized around urate burden, inflammatory/oxidative stress, neurovascular function, and tissue integrity. The proposed TCM-biomedical mappings are translational analogies rather than established molecular equivalences, and the framework is not evidence that integrated Chinese and Western medicine is already effective for HUAED. HUAED-specific mechanistic studies and prospective trials are required.

## 1 Introduction

Hyperuricemia (HUA) and erectile dysfunction (ED) frequently coexist in men with metabolic disorders. Available human and experimental evidence points to overlapping metabolic, inflammatory, oxidative, and vascular abnormalities, yet no single causal cascade has been established. In the clinical component of their study, Shen et al. (1) found that in two clinical datasets, each 100 μmol/L increase in serum urate was associated with higher odds of ED (Study I: OR 2.522, 95% CI 1.068–5.958; Study II: OR 2.951, 95% CI 1.560–5.584), rather than representing a categorical comparison between hyperuricemic and normouricemic participants; the same study employed Uox-knockout rats and cellular experiments to examine urate-dependent stabilization of myosin light chain kinase (MLCK). Other work supports systemic endothelial dysfunction and macrophage-mediated inflammatory complications of HUA, but does not directly demonstrate penile fibrosis or arterial ED (2, 3). This review therefore treats the proposed sequence from metabolic dysregulation to inflammation, neurovascular dysfunction, cavernosal hypercontractility, and possible structural remodeling as a working model. TCM concepts such as damp-heat, phlegm, stasis, and collateral obstruction are used only as heuristic translational analogies to systemic metabolic and inflammatory states (4–7), not as one-to-one molecular equivalents. Evidence implicating the NLRP3, JAK/STAT, and MAPK pathways in HUAED is predominantly preclinical and derives partly from related experiments by the same research group using similar rat models and Quyuhuatanerxian decoction (QYHT); independent replication is therefore needed (5, 6).

The current challenge in managing HUAED is that most available interventions target only single components of a multifactorial pathological network. Although urate-lowering therapy (ULT) effectively reduces serum uric acid (SUA) levels, its impact on erectile function-related outcomes remains unclear, particularly as HUA is increasingly recognized as a systemic metabolic-inflammatory state involving oxidative stress, vascular injury, and endothelial dysfunction, and the benefits of ULT beyond serum urate reduction are still incompletely defined (8–10). While phosphodiesterase type 5 (PDE5) inhibitors remain first-line pharmacotherapy for ED (11, 12), they primarily improve erectile symptoms and may be less effective in settings of persistent endothelial dysfunction and cavernosal structural damage, with limited effects on broader pathological processes potentially involved in HUAED, including inflammation, oxidative stress, endothelial dysfunction, and cavernosal structural remodeling (13–16). Therefore, by integrating epidemiological, clinical phenotypic, genetic, and experimental evidence, this paper proposes a phenotype-stratified framework for integrated Traditional Chinese and Western medicine in HUAED. This framework is intended to offer a translational hypothesis for future research rather than direct proof of established efficacy in this specific condition.

We searched PubMed and the Web of Science Core Collection from inception to August 10, 2026, using terms for HUA, ED, TCM, acupuncture, integrative medicine, and conventional treatment, without a language restriction. The PubMed strategy combined controlled vocabulary with Title/Abstract and free-text terms, whereas Web of Science used Topic fields; the resulting counts are therefore database-specific and not numerically interchangeable. The six Web of Science records reflect differences in database coverage, indexing, and field mapping rather than application of a more restrictive eligibility threshold. The searches identified 2,453 records (PubMed, 2,447; Web of Science, 6). After removal of six duplicates, 2,447 unique records underwent title/abstract screening. Of these, 2,424 were excluded because they failed one or more prespecified eligibility criteria, including lack of a direct HUA-ED focus, assessment of unrelated diseases or outcomes, absence of a relevant mechanistic or clinical link, or irrelevance to the conventional, TCM, or integrative intervention domains of this review. Twenty-three core records were retained from the structured search; one Chinese-language practice guideline was eligible because no language restriction was applied. Because direct HUAED evidence was sparse, backward/forward citation tracking and author-knowledge supplementation were used only for studies that directly supported a principal claim, clarified a mechanistic gap when HUAED-specific evidence was unavailable, or provided guideline/methodological context. Initial title/abstract screening was performed by SH. All 23 core records retained from the structured search were independently verified against the eligibility criteria by YNF. Disagreements were resolved through discussion and consensus; any unresolved disagreement was adjudicated by YFZ. Each retained citation was classified by retrieval source and evidence context (HUAED-specific, HUA-only, ED-only, or non-penile/indirect). **Supplementary Table S1** reports the database counts, screening summary, retained records, and eligibility criteria; **Supplementary Appendix S1** provides the complete search strings and keyword framework. **Supplementary Table S2** provides the final citation-evidence audit. Chinese-language databases (e.g., CNKI, Wanfang, and SinoMed) were not searched because the predefined structured component was limited to PubMed and Web of Science and the review was not intended to be exhaustive; this decision may under-represent Chinese-language TCM evidence and is treated as a specific limitation. Consistent with a narrative review, we did not apply a PRISMA flow diagram or formal certainty grading; instead, we performed a structured domain-based appraisal of study design, population or model, exposure/intervention, comparator, quantitative findings, directness, and principal limitations, summarized in **Tables 1**–**3**.

**Table 1:** Structured appraisal of epidemiological evidence linking gout, categorical hyperuricemia, and continuous serum urate with erectile dysfunction.

Exposure / reference	Design and sample	Comparison	Effect estimate	Directness	Key limitation
Gout (18)	Systematic review/meta-analysis	Gout vs non-gout	Unadjusted pooled OR 1.44 (95% CI 1.20–1.72); cohort subgroup OR 1.20 (1.10–1.32)	Clinical; gout, not isolated HUA	Residual confounding; cross-sectional subgroup nonsignificant
Incident gout (19)	Population-based cohort; 9,653 gout and 38,218 controls	Incident gout vs matched controls	193 vs 132 ED events/10,000 person-years; adjusted HR 1.31 (1.24–1.40)	Clinical; primary cohort	Observational; gout-related inflammation and treatment may confound
Categorical HUA (24)	Systematic review/meta-analysis; 5 cohort + 6 cross-sectional studies; 454,510 participants	HUA vs non-HUA	Pooled OR 1.59 (1.29–1.97)	Clinical; mixed definitions	High heterogeneity; mixed designs and coding
Continuous SUA (1)	Two clinical datasets: Study I, 33 ED/43 controls; Study II, 94 ED/67 controls	Per 100 μmol/L higher SUA	Study I OR 2.522 (1.068–5.958); Study II OR 2.951 (1.560–5.584)	Clinical; continuous exposure	Selected younger populations; not a categorical HUA comparison
Categorical HUA (21)	NHANES cross-sectional analysis; n=3,810	HUA vs non-HUA	No significant association after multivariable adjustment	Clinical; population survey	Cross-sectional; residual confounding and definition dependence
Genetically predicted SUA (22)	Two-sample Mendelian randomization	Genetic instrument for SUA	No confirmed causal effect on ED	Genetic causal inference	Instrument assumptions; not equivalent to treatment effect
ULT in HUA (24)	Pooled observational evidence	No ULT vs ULT, as implied by authors’ direction	OR 1.27 (1.14–1.41); reciprocal 0.79	Indirect treatment evidence	Coding/reporting ambiguity; no randomized HUAED trial

**Table 2:** Mechanistic evidence matrix according to disease specificity and experimental context.

Pathway / reference	Model / tissue	Principal finding	Evidence context	Interpretive constraint
Urate–MLCK/MLC2 (1)	Uox-knockout rat; cavernosal smooth-muscle cells	UA–MLCK interaction, reduced NEDD4L-mediated ubiquitination, MLCK stabilization, p-MLC2 ↑, impaired erection	Direct HUAED-specific	Preclinical; human target engagement not shown
Early NO signaling (1)	20-week Uox-knockout rat penile tissue	NO metabolites ↔; p-eNOS/eNOS ↓; p-nNOS/nNOS ↑	Direct HUAED-specific	Phosphorylation ratios do not establish neural function
nNOS/eNOS and ROS (36)	Chemically induced HUA rat penile tissue	ROS ↑; nNOS/eNOS-related signals ↓; erectile measures impaired	Direct HUAED-specific	Different model and exposure duration from Uox-knockout study
MYPT1/MLCP and ROCK (1, 49)	Uox-knockout HUAED vs hypertensive ED	MYPT1/p-MYPT1 unchanged early in HUAED; ROCK1/2 increased in hypertensive ED	Direct HUAED negative finding + other-ED indirect evidence	No direct HUAED proof of a sequential ROCK/MLCP mechanism
Nrf2/HO-1 (39–42)	High-UA endothelial cells; diabetic/hypertensive ED; ox-LDL endothelial model	Model-dependent antioxidant or protective responses	Mostly non-penile or other-ED indirect evidence	No direct penile Nrf2 experiment in HUAED
NLRP3/JAK-STAT (5, 6, 38)	Related HUAED rat studies; intestinal HUA model	Inflammatory/oxidative markers and pathway expression changed	Limited HUAED-specific cluster + non-penile indirect evidence	Overlapping group/protocol; independence unclear; target engagement incomplete
Fibrosis/remodeling (1, 49)	Late Uox-knockout rat and hypertensive ED	Muscle-fiber loss at 43 weeks; remodeling plausible	Preclinical and partly indirect	No direct human HUAED fibrosis evidence

**Table 3:** Quantitative and comparative appraisal of treatment evidence relevant to HUAED.

Evidence category / reference	Design and n	Population or model	Intervention, dose, duration	Comparator	Principal effect and safety
HUAED-specific: ULT/MLCK (1)	Preclinical; WT n=6, Uox-KO n=6; intervention groups n=5	Uox-knockout rats	Febuxostat, benzbromarone, compound 3170, or ML-7; weeks 16–20	Untreated Uox-KO and WT	Erectile measures improved; adverse-event assessment not reported; mechanism not isolated to urate lowering
HUAED-specific: febuxostat (36)	Preclinical; group n not reported in abstract	Chemically induced HUA rats	Febuxostat 5 mg/kg/day for 4 weeks	HUA model and normal controls	Partial restoration of erectile and nNOS/eNOS-related measures; animal safety data insufficient
HUAED-specific: QYHT cluster (5, 6)	Human exploratory n=30; QYHT rat study n=60; monomer experiments n=60 each	HUAED patients/rat models	QYHT 100 mL three times/day for 12 weeks; rats 9.45–37.8 mg/kg for 28 days; linoleyl acetate 40–160 mg/kg; mandenol 8–32 mg/kg	Healthy/model controls; no placebo-controlled human erectile endpoint	Biomarker and preclinical erectile signals; adverse-event counts not adequately reported; related groups/protocols and unclear cohort independence
HUA only: Wu-Ling San / Yin Chen Wu-Ling San (55)	Randomized double-blind placebo-controlled; n=60, three arms	Adults with SUA >8 mg/dL; ED not assessed	Two formulas for 4 weeks; outcomes at weeks 2, 4, and 8; dose not reported in abstract	Placebo	No significant difference in target SUA <6 mg/dL at week 4; week-8 SUA 8.1 vs 9.1 mg/dL for Yin Chen Wu-Ling arm (P = .034); described as short-term relatively safe, but AE counts unavailable in abstract
ED only: CHM + tadalafil (13)	Meta-analysis of 11 RCTs; n=903	ED; HUA not required	Heterogeneous CHM + tadalafil 5 or 10 mg schedules; 4–12 weeks	Tadalafil alone	IIEF-5 WMD 2.49 (2.213–2.767) after sensitivity analysis; SEP-Q2 RR 1.191 (1.025–1.384); AE RR 0.83 (0.36–1.88), 28 aggregate events; limited post-treatment follow-up
ED only: Hongjing I (58)	Multicenter double-blind placebo-controlled RCT; 122 completers (61/arm)	Mild-to-moderate ED with qi deficiency/blood stasis; HUA not required	Hongjing I granules, one packet twice daily, 8 weeks	Matched placebo	IIEF-EF change 7.80 ± 3.25 vs 3.33 ± 3.90 (P<.001); no AEs reported; final visit at week 12
ED only: acupuncture (59, 60)	Systematic reviews/meta-analyses of RCTs	Mixed ED etiologies; HUA not required	Heterogeneous acupuncture protocols and durations	Sham, medication, or other controls	Possible symptom benefit; certainty and adverse-event reporting limited; not HUAED-specific

## 2 Epidemiological and clinical phenotypic characteristics

### 2.1 Current epidemiological evidence for HUA and ED: association, limitations, and heterogeneity

Current epidemiological evidence supporting an association between HUA and ED remains limited and heterogeneous. The available literature is composed mainly of cross-sectional studies, several cohort studies, and pooled analyses, and the reported associations are not fully consistent across different populations and study designs. In addition, variations in age distribution, metabolic comorbidity burden, definitions of HUA, ED assessment tools, and covariate adjustment strategies may all contribute to the observed heterogeneity. Therefore, the existing epidemiological evidence is better interpreted as suggesting a possible association rather than establishing a uniform, robust, or causal relationship.

Several large studies support an association between gout or HUA and ED, although effect estimates vary. Meta-analyses reported higher odds of ED among men with gout (17, 18). In the primary population-based cohort now cited as reference 19, 9,653 men with incident gout were compared with 38,218 matched controls; ED incidence was 193 versus 132 per 10,000 person-years, and the adjusted hazard ratio was 1.31 (95% CI 1.24–1.40). A body mass index-matched population-based study similarly reported an adjusted hazard ratio of 1.15 (95% CI 1.09-1.21) (20). Conversely, an NHANES analysis of 3,810 US adults found no significant association after adjustment (21). Mendelian-randomization analyses have not confirmed a causal effect of genetically predicted serum urate on ED (22). A prevalence meta-analysis estimated ED in 33% of patients with HUA (95% CI 13%–52%) (23). Reference 24 separately reported higher ED odds for categorical HUA versus non-HUA (pooled OR 1.59, 95% CI 1.29–1.97). For ULT, the paper presented OR 1.27 (95% CI 1.14–1.41) in the direction of no ULT versus ULT; its reciprocal is 0.79, corresponding to approximately 21% lower odds among ULT users—not a 27% reduction. Because the contributing data were observational and the original comparison coding was not transparently reported, this estimate does not establish a preventive or therapeutic effect of ULT on ED.

### 2.2 HUAED phenotypic stratification factor

HUAED is unlikely to represent a homogeneous phenotype; age, metabolic status, and hormonal factors may modify the observed association. In the human clinical component of Shen et al. (1), each 100 μmol/L increase in serum urate was associated with higher ED odds (Study I: OR 2.522, 95% CI 1.068–5.958; Study II: OR 2.951, 95% CI 1.560–5.584). An adolescent obesity study linked excess urate to metabolic syndrome but did not evaluate ED, thereby providing only indirect metabolic context (25). In men with newly diagnosed type 2 diabetes, HUA independently predicted incident ED, as did age over 60 years (26). In gout, HOMA-IR independently predicted ED (adjusted OR 1.62, 95% CI 1.03–2.82) (27); studies of urate and insulin resistance offer indirect metabolic evidence but do not by themselves establish the HOMA-IR–ED association (28–30). Dysglycemia and dyslipidemia also correlate with poorer erectile function (31, 32). TCM constitution studies describe broad disease associations but do not establish a specific damp-heat HUAED phenotype, and any such mapping should therefore be regarded as exploratory (33). Hormonal evidence is likewise inconsistent: one population study found a positive association between urate and free androgen index but lower urate among men with ED (34), whereas a bidirectional analysis using UK Biobank data linked genetically predicted higher urate with lower sex hormone-binding globulin and testosterone (35). Impaired thyroid-hormone sensitivity has been associated with HUA, with cholesterol as a partial mediator, but this does not directly establish an ED mechanism (2). These data support age, metabolic-inflammatory status, and hormonal profile as candidate stratification variables rather than validated treatment categories.

## 3 Integration of pathological mechanisms: metabolism-inflammation-angiogenic remodeling

**Figure 1** presents a hypothesis-generating framework for hyperuricemia-associated erectile dysfunction (HUAED), with explicit labels distinguishing HUAED-supported findings from indirect or unvalidated links. Elevated urate may promote mitochondrial reactive oxygen species (ROS), attenuate nuclear factor erythroid 2-related factor 2 (Nrf2)/heme oxygenase-1 (HO-1) antioxidant defense, and enhance NLRP3 inflammasome-associated inflammation. However, the Nrf2/HO-1 and NLRP3 components are primarily supported by indirect endothelial or non-HUAED evidence, and direct penile evidence in HUAED remains limited. Erection is initiated by cavernous-nerve neuronal nitric oxide synthase (nNOS)-derived nitric oxide (NO) and maintained by endothelial nitric oxide synthase (eNOS)-derived NO through cyclic guanosine monophosphate (cGMP) signaling; HUAED-specific evidence for neural impairment remains limited. In cavernosal smooth muscle, the urate–myosin light-chain kinase/myosin regulatory light-chain 2 (MLCK/MLC2) pathway may increase contractile tone and impair relaxation. The proposed transition from functional impairment to collagen deposition, vascular fibrosis, and tissue remodeling is explicitly presented as a testable hypothesis rather than a directly demonstrated feature of human HUAED. The TCM concept of damp-heat is included only as a heuristic translational construct and does not represent an established molecular equivalence.

**Figure 1:**
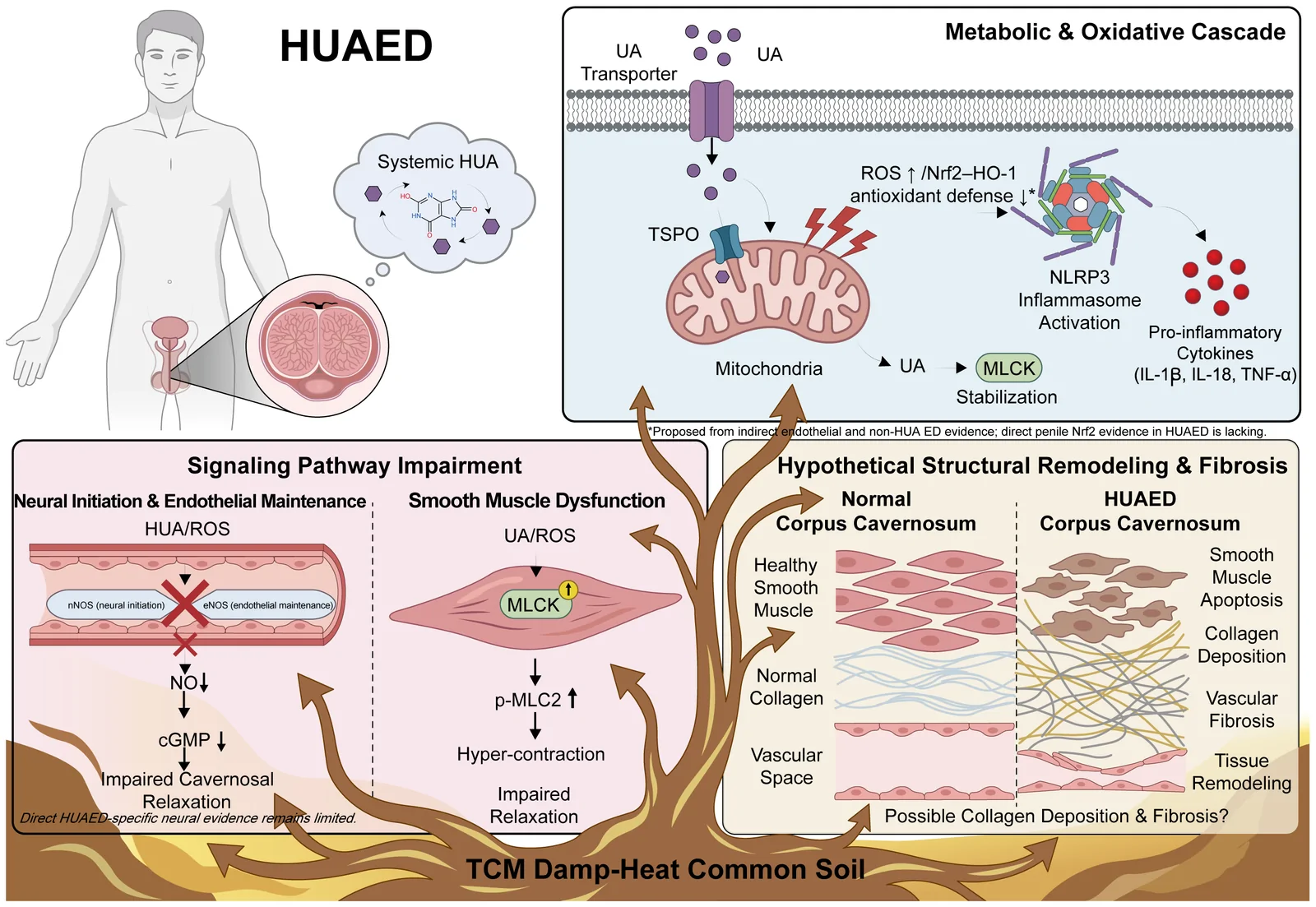
A hypothesis-generating framework linking the shared damp-heat concept in TCM with putative metabolic, inflammatory, neurovascular, and structural pathways in HUAED.

### 3.1 Abnormal uric acid metabolism and the oxidative stress cascade

The evidence summarized in this section is predominantly preclinical and supports mechanistic plausibility rather than a clinically established causal pathway in HUAED. Within this evidentiary context, HUA is associated with oxidative stress through multiple mechanisms relevant to ED pathophysiology. For the TCM-related interpretation in this section, the biomedical–TCM correspondence is used as a heuristic translational framework rather than a direct biological equivalence. From this perspective, long-term elevation of UA and accumulation of related metabolic products are discussed in relation to the TCM notion of “internal obstruction of turbid dampness”, while accompanying microcirculatory abnormalities and tissue stress provide a translational context for the progression from systemic metabolic imbalance to localized tissue dysfunction.

Mechanistic findings are not uniform across experimental models. In the chemically induced hyperuricemic rat model, penile ROS increased and eNOS, phosphorylated eNOS, and nNOS signals decreased (36). By contrast, at the early dysfunctional stage of the Uox-knockout model, NO metabolites were unchanged, the p-eNOS/eNOS ratio decreased, the p-nNOS/nNOS ratio increased, MYPT1 and p-MYPT1 did not change significantly, and MLCK/p-MLC2 activity increased (1). High urate also reduced endothelial NO and increased inflammatory mediators *in vitro* (37). In a hyperuricemic mouse model, the 18-kDa translocator protein (TSPO) was implicated in mitochondrial depolarization, ROS generation, NLRP3 activation, and intestinal-barrier injury (38). These discrepancies may reflect spontaneous versus chemically induced hyperuricemia, exposure duration, disease stage, tissue sampling, and measurement method. They support potentially parallel, stage-dependent contractile and neurovascular mechanisms rather than a uniform linear cascade. Nrf2 evidence adds an important but still indirect layer. Simvastatin activated Nrf2 and reduced high-urate-induced oxidative and inflammatory injury in vascular endothelial cells (39). In non-HUA ED models, dimethyl fumarate improved diabetic ED through Nrf2-mediated suppression of penile endothelial oxidative stress (40), and fatty-acid-synthase inhibition improved hypertension-associated ED through Nrf2/HO-1 activation and reduced NLRP3-dependent pyroptosis (41). Conversely, suitable urate concentrations protected ox-LDL-exposed endothelial cells through Keap1-Nrf2-antioxidant response element signaling (42). Thus, urate-Nrf2 effects appear concentration-, compartment-, and model-dependent; direct Nrf2 studies in HUAED penile tissue are still lacking.

### 3.2 Neurovascular nNOS/eNOS/NO/sGC/cGMP signaling and ED

Penile erection is a neurovascular event rather than an exclusively endothelial process. Cavernous-nerve and nonadrenergic noncholinergic signaling initiate erection through nNOS-derived NO; endothelial shear stress then recruits eNOS-derived NO to amplify and maintain sGC/cGMP-dependent cavernosal relaxation. The reductions in nNOS- and eNOS-related signals in the induced-HUA rat model (36) contrast with the increased p-nNOS/nNOS ratio, reduced p-eNOS/eNOS ratio, and unchanged NO metabolites in early Uox-knockout ED (1); neither model resolves cavernous-nerve electrophysiology or the relative neural and endothelial contributions. Systemic flow-mediated dilation is impaired in HUA, but this finding alone does not establish penile endothelial dysfunction (37). Preclinical studies further indicate that Akt/eNOS signaling can modify HUA-associated endothelial injury (43), whereas acute high-urate exposure did not impair endothelium-dependent relaxation in several large arteries of aged rats (44). These discrepancies may reflect differences in exposure duration, urate concentration, vascular bed, animal age, and model design. The current evidence therefore supports a neurovascular working model in which oxidative stress may weaken neural initiation and endothelial maintenance of erection, but HUAED-specific cavernous-nerve electrophysiology, nNOS localization, and human penile vascular data are still needed. The TCM terminology used in this section serves as a translational analogy to impaired perfusion and tissue support, not as a claim of molecular equivalence.

### 3.3 NLRP3 inflammasome activation and tissue damage

NLRP3 inflammasome activation may represent an inflammatory link between HUA and ED, though direct evidence in HUAED remains limited. Urate-related signals and ROS can promote inflammasome assembly, caspase-1 activation, and IL-1β/IL-18 maturation in several experimental systems (3). Oxidative stress has been implicated in urate-related vascular remodeling (45), and inflammasome-driven vascular inflammation may amplify endothelial injury (46). Reference 5 combined a small exploratory human biomarker cohort (30 participants; 10 per group; no placebo-controlled erectile-function endpoint) with HUAED rat experiments (60 rats; 12 per group), and reported lower NLRP3-related, oxidative, and inflammatory markers after QYHT. Reference 6, from an overlapping investigator group, tested two candidate monomers in separately described HUAED rat experiments using closely similar induction and fecal-transplantation procedures. The publications do not explicitly establish whether any animals or samples overlapped; cohort independence therefore cannot be assumed, and the two reports should be viewed as a related evidence cluster requiring external replication rather than independent confirmation (5, 6). Evidence from multiple sclerosis (47), intestinal-barrier models (38), and a dual NLRP3/URAT1 inhibitor in gouty arthritis (48) is mechanistically informative but indirect for HUAED. Within the integrative framework, TCM damp-heat terminology is used as a heuristic analogy to sustained inflammatory burden rather than a definitive molecular representation of NLRP3 activity.

### 3.4 Smooth muscle dysfunction and fibrotic remodeling

Cavernosal smooth-muscle tone depends on the balance between MLCK-mediated MLC2 phosphorylation and myosin light chain phosphatase (MLCP)-mediated dephosphorylation. In Uox-knockout rats, urate entered cavernosal smooth-muscle cells, interacted with MLCK at N803, impaired ubiquitination mediated by NEDD4-like E3 ubiquitin protein ligase (NEDD4L), stabilized MLCK, and increased MLC2 phosphorylation (1). A complementary calcium-sensitization pathway involves RhoA/ROCK-dependent phosphorylation of MYPT1 and inhibition of MLCP. Increased ROCK1/ROCK2 and impaired relaxation have been observed in hypertension-associated ED, which is indirect rather than HUA-specific evidence (49). At the early ED stage of the Uox-knockout model, MYPT1 and p-MYPT1 were unchanged despite increased MLCK/p-MLC2 activity (1). The urate–MLCK/MLC2 axis and neurovascular injury should therefore be considered potentially parallel and stage-dependent; a sequential transition to RhoA/ROCK–MYPT1/MLCP dysregulation has not been demonstrated in HUAED. In the Uox-knockout model, overt muscle-fiber loss was evident at 43 weeks but not 20 weeks, suggesting time-dependent structural progression (1). Direct human evidence that HUA causes cavernosal fibrosis remains absent; consequently, progression from persistent hypercontractility and hypoperfusion to collagen remodeling should be treated as a testable hypothesis, not an established human pathway. TCM descriptions of stasis and collateral obstruction are used only as stage-oriented translational analogies.

### 3.5 Mechanism basis of the three-level intervention model

Level 0 (“TCM Direct Intervention”) is a cross-level research domain rather than a fourth sequential stage. **Figure 2** summarizes this three-tiered, hypothesis-generating intervention model. Levels 1–3 refer respectively to upstream urate/metabolic burden, intermediate inflammatory-oxidative-neurovascular dysfunction, and downstream erectile or structural outcomes. Candidate TCM interventions may be studied across these levels, but their traditional principles should not be equated with specific molecular targets. At Level 1, urate production, excretion, metabolic status, and oxidative burden are candidate outcomes. At Level 2, Nrf2/HO-1, NLRP3, neurovascular NO signaling, and endothelial function are candidate mechanistic outcomes. At Level 3, erectile hemodynamics, cavernosal contractile balance, and tissue integrity are candidate functional or structural outcomes. Evidence-based ED and HUA care remains the comparator, while TCM approaches require disease-specific evaluation.

**Figure 2:**
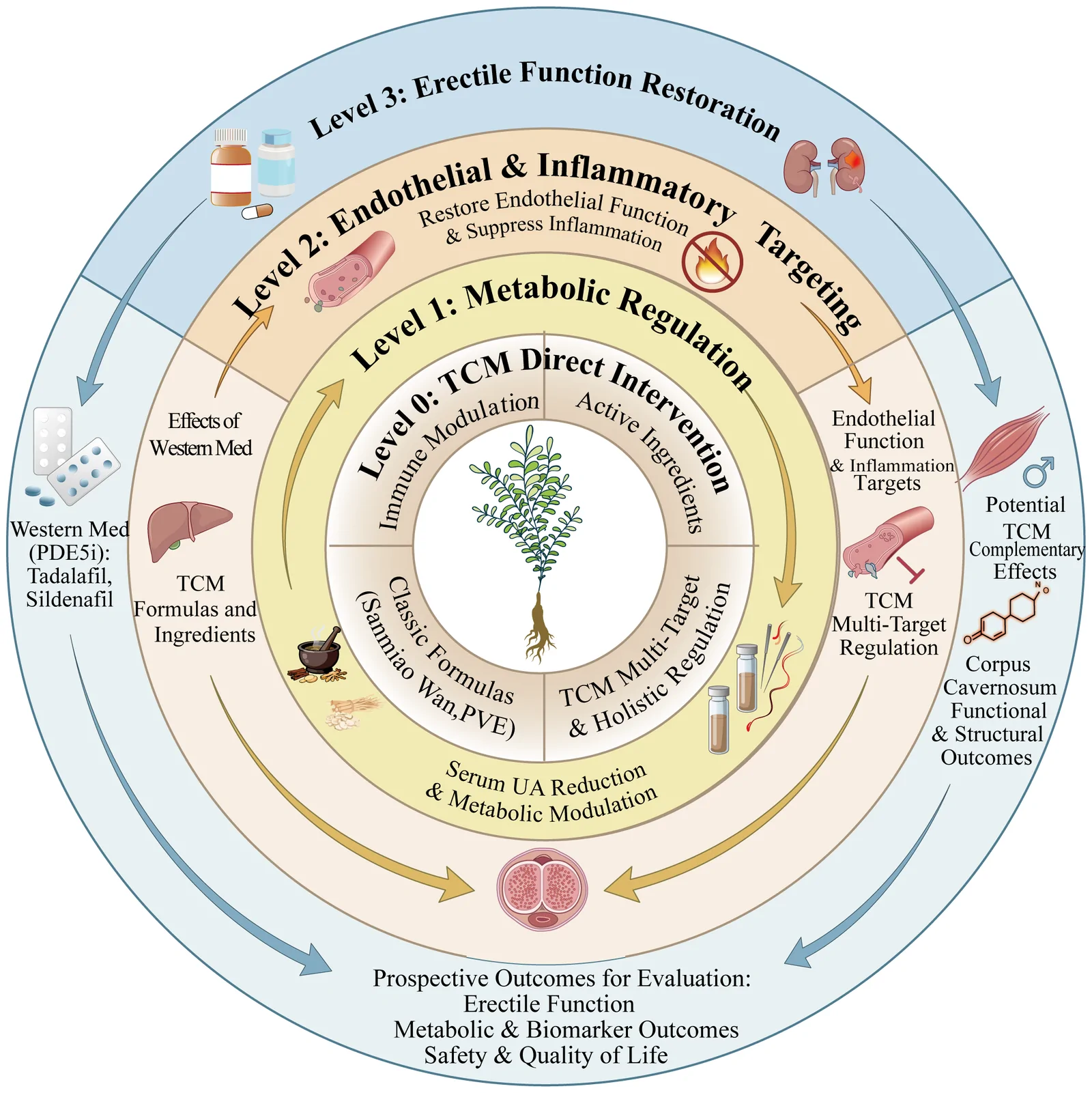
Diagram of a three-tiered collaborative intervention model integrating western and TCM.

## 4 Evidence-based evaluation of treatment strategies

### 4.1 Western medicine treatment: precision use of ULT and PDE5 inhibitor therapy

#### 4.1.1 Therapeutic endpoints and the “missing middle”

ULT and ED therapies address different clinical targets. ULT should be used according to established gout/HUA indications, whereas ED treatment follows guideline-based evaluation and shared decision-making (12). Between serum-urate control and erectile symptom relief lies a hypothesized “missing middle” comprising chronic inflammation, oxidative stress, neurovascular injury, cavernosal hypercontractility, and possible tissue remodeling. These intermediate processes are plausible research targets but are not yet validated HUAED treatment indications.

#### 4.1.2 sGC biology and limitations of PDE5 inhibition

PDE5 inhibitors reduce cGMP degradation; they do not generate cGMP. Their effect therefore depends on adequate neural/endothelial NO and reduced heme-containing sGC. Oxidative stress can oxidize or remove the sGC heme group and reduce NO responsiveness. sGC stimulators and activators are consequently mechanism-based research candidates, but no HUAED-specific efficacy data currently support their clinical use.

#### 4.1.3 Concentration-dependent urate redox effects and Nrf2

Urate is not uniformly pro-oxidant. Extracellular urate can act as an antioxidant, whereas intracellular urate may promote NADPH-oxidase and mitochondrial ROS. Simvastatin activated Nrf2 in high-urate-exposed endothelial cells (39), while Nrf2 activation improved diabetic and hypertension-associated ED in animal models (40, 41). Suitable urate concentrations also protected ox-LDL-exposed endothelial cells through Keap1-Nrf2-antioxidant response element signaling (42). These findings establish biological plausibility and concentration dependence, not a validated Nrf2 mechanism in HUAED. The lower serum-urate boundary for erectile benefit or harm is unknown.

#### 4.1.4 Xanthine oxidase inhibition versus urate lowering

Xanthine oxidase (XO) inhibitors simultaneously reduce serum urate and XO-derived ROS, so endothelial improvement with febuxostat or allopurinol cannot be attributed to urate lowering alone. In the Shen et al. animal experiment, benzbromarone, febuxostat, and compound 3170 improved erectile-function-related measures under hyperuricemic conditions (1). Because the study was not designed to isolate uricosuric from XO-inhibitory effects, the causal mediator remains unresolved. A separate hyperuricemic rat study reported that febuxostat partly restored penile eNOS/nNOS-related signals and erectile measures (36).

#### 4.1.5 Clinical ULT evidence and drug-specific uncertainty

Clinical evidence that ULT prevents or treats ED is inconclusive. The pooled estimate reported in reference 24 cannot be interpreted as a risk reduction without a defined comparison direction. In a propensity-score-matched cohort, febuxostat was associated with higher incident ED risk than allopurinol, particularly among men aged 19–64 years (8). The apparent divergence between animal and observational evidence may reflect species, dose, timing, comorbidity, confounding by indication, and treatment-selection bias. Current evidence therefore supports individualized ULT based on guideline indications and safety monitoring, not ED-specific urate thresholds or a universal preference for or against one agent.

### 4.2 Traditional Chinese medicine treatment: mechanism of action of classic formulas and active ingredients

Recent reviews summarize possible effects of TCM interventions on urate production, urate transport, inflammation, oxidative stress, and gut–kidney interactions, but most compound-specific evidence remains preclinical (50, 51). Reference 5 included an exploratory, non-placebo-controlled human biomarker cohort (n=30; 12-week QYHT exposure) and a 60-rat HUAED experiment (12 per group; QYHT 9.45, 18.9, or 37.8 mg/kg for 28 days). Reference 6 tested linoleyl acetate (40, 80, or 160 mg/kg) and mandenol (8, 16, or 32 mg/kg) in similarly constructed HUAED rat experiments, with n=3 reported for several assay-level comparisons. Because the investigator groups, induction methods, and fecal-transfer procedures overlap and sample independence is not explicitly documented, these reports provide a related preclinical evidence cluster rather than independent replication (5, 6). Sanmiao Wan altered metabolic profiles in hyperuricemic rats (52), and berberine reduced apoptosis in diabetic ED rats (53); neither study directly validates treatment of HUAED. Paeonia veitchii reduced urate in experimental models through XO and renal-transport mechanisms (54). A placebo-controlled trial of Yin Chen Wu Ling San reported a modest serum-urate reduction at eight weeks (55), while a real-world study found that CHM users with HUA had a lower subsequent gouty-arthritis risk and a liver-injury risk comparable to benzbromarone (56). These HUA outcomes should not be generalized to ED. Hongjing I granule improved selected outcomes in mild-to-moderate ED in a randomized trial (58), but HUA was not an enrollment criterion. Overall, these data justify prospective HUAED studies with standardized products and endpoints, not current claims of efficacy for HUAED.

### 4.3 Clinical evidence relevant to the integrated management of HUAED

Clinical evidence directly evaluating integrated treatment in HUAED is not yet available. The CHM-plus-tadalafil meta-analysis included 11 randomized trials and 903 participants (451 combination; 452 tadalafil alone) treated for 4–12 weeks (13). After sensitivity analysis, the pooled IIEF-5 mean difference was 2.49 points (95% CI 2.213–2.767), and the pooled SEP-Q2 response was RR 1.191 (95% CI 1.025–1.384). Seven studies mentioned adverse events; six provided arm-specific data, yielding RR 0.83 (95% CI 0.36–1.88). Across reports, 28 events were described (10 mild headaches, 7 flushing events, 5 dyspepsia events, 2 shoulder/back pain events, 2 mild pharyngeal pain events, 1 myalgia event, and 1 nasal-congestion event), but aggregate reporting prevents reliable arm-specific frequency calculation. Follow-up generally ended with the 4–12-week treatment period; only two studies reported approximately 4 weeks after withdrawal. These ED-only data support, at most, a possible incremental benefit; heterogeneous formulas, limited blinding, short follow-up, and imprecise safety estimates preclude conclusions about HUAED or pharmacological synergy (13). In the Hongjing I trial, 122 men with mild-to-moderate ED completed an 8-week randomized, double-blind, placebo-controlled intervention; mean IIEF-EF change was 7.80 ± 3.25 versus 3.33 ± 3.90 (P<0.001), and no adverse events were reported through the 12-week final visit, but HUA was not an eligibility criterion (58). Acupuncture systematic reviews and meta-analyses report possible ED benefits, although certainty remains limited and the included populations were not restricted to HUAED (59, 60). Preclinical HUAED studies of QYHT (5, 6), hyperuricemic animal studies of ULT (1, 36), and studies of TCM interventions in HUA or non-HUA ED populations provide indirect mechanistic context. They do not establish clinical synergy. Therefore, combined interventions should be tested in prospective, phenotype-defined studies against guideline-based care, with prespecified erectile, metabolic, neurovascular, and safety outcomes.

This diagram presents a hypothesis-generating framework for potential integrated TCM and Western medicine interventions in HUAED. The framework contains a foundational “Level 0: TCM Direct Intervention” domain together with three formal sequential levels corresponding to metabolic regulation, endothelial/inflammatory targeting, and erectile-function restoration. Level 0 should not be interpreted as a fourth stage in the prevention-regulation-repair sequence. Rather, it represents a cross-level TCM intervention foundation comprising classic formulas, active ingredients, proposed immune-related effects, and multi-target holistic regulation that may potentially interact with mechanisms represented at Levels 1-3.

Level 1, “Metabolic Regulation,” represents the upstream metabolic research domain and focuses on SUA burden and associated metabolic abnormalities. Candidate research targets include purine metabolism, amino acid metabolism, urate production and excretion, gut microbiota, and related metabolic networks. ULT and TCM-related approaches are illustrated as candidate interventions whose effects on these metabolic processes require further evaluation. Level 2, “Endothelial & Inflammatory Targeting,” focuses on candidate intermediate mechanisms linking persistent HUA with ED, including endothelial dysfunction, oxidative stress, chronic inflammation, impaired endothelial nitric oxide synthase (eNOS)/NO/cGMP signaling, ROS accumulation, NLRP3 inflammasome activation, and inflammatory pathways involving JAK/STAT and MAPK. The interventions illustrated at this level represent candidate approaches for mechanistic evaluation rather than established HUAED treatments. Level 3, “Erectile Function Restoration”, represents the downstream functional and structural research domain, including cGMP-mediated smooth muscle relaxation, penile hemodynamics, smooth muscle integrity, apoptosis, fibrosis, extracellular-matrix remodeling, hormonal pathways, and preservation of corpus cavernosum structure. PDE5 inhibitor therapy and candidate TCM interventions are shown as strategies that may be prospectively evaluated individually or in combination rather than as a validated HUAED treatment regimen. The arrows connecting these domains illustrate hypothesized interactions among metabolic abnormalities, inflammation and oxidative stress, endothelial dysfunction, and erectile or structural impairment. The outcomes shown at the bottom of the figure should be interpreted as candidate endpoints for prospective evaluation rather than demonstrated clinical benefits. HUAED-specific mechanistic studies and prospective randomized controlled trials are required to validate these relationships and candidate interventions.

## 5 Clinical translation pathways and implementation strategies

### 5.1 Suggestions for optimizing the endpoint indicator system

Future HUAED studies should pair validated erectile-function outcomes, including IIEF-5 and Sexual Encounter Profile measures, with objective biomarkers. Four domains are proposed: metabolic measures such as serum urate, HOMA-IR, glycated hemoglobin, and lipid profile (27, 31); inflammatory and oxidative-stress measures such as IL-1β, IL-18, malondialdehyde, and superoxide dismutase (5, 6, 38); neurovascular measures such as penile Doppler parameters, NO-related markers, and endothelial-function assessments (36, 37); and hormonal measures such as total testosterone, sex hormone-binding globulin, and free androgen index (26, 34, 35). In animal studies, MLCK/MLC2 phosphorylation, MYPT1/MLCP activity, and the smooth-muscle-to-collagen ratio may serve as mechanistic endpoints (1, 49). Non-invasive fibrosis imaging or circulating remodeling markers could be explored, but their validity for HUAED remains unestablished. A TCM-constitution score, if used, should be prospectively validated and analyzed separately from biomedical biomarkers.

### 5.2 Proposed phenotype-informed research framework

The following metabolic-inflammatory, hormonal, and neurovascular domains are overlapping research features rather than mutually exclusive strata or treatment algorithms. A metabolic stratum may include insulin resistance, diabetes, dyslipidemia, and elevated urate; HOMA-IR independently predicted ED in gout (27). A hormonal/age stratum may include older men or men with clinically confirmed hypogonadism, but testosterone therapy must follow established diagnostic and safety guidance rather than a fixed threshold proposed by this review (26). In the human clinical component of Shen et al. (1), each 100 μmol/L increase in serum urate was associated with higher ED odds (Study I: OR 2.522, 95% CI 1.068–5.958; Study II: OR 2.951, 95% CI 1.560–5.584); this association supports risk assessment, not pharmacological ULT solely for ED prevention. A neurovascular stratum may be considered in diabetes, neuropathic symptoms, or impaired vascular function because nNOS-dependent neural initiation and eNOS-dependent endothelial maintenance are both relevant to erection (32, 36). QYHT findings in HUAED rats (5, 6), Sanmiao Wan findings in hyperuricemic rats (52), and CHM-plus-tadalafil findings in broader ED populations (13) remain indirect or preliminary for phenotype-specific HUAED care. Observationally, febuxostat was associated with higher ED incidence than allopurinol in some gout populations (8), whereas animal data suggest potential benefit (1, 36). Accordingly, future trials should stratify by age, metabolic status, neural/vascular involvement, inflammatory burden, and treatment exposure while using guideline-based care as the comparator. No HUAED-specific serum-urate initiation target or lower-benefit threshold has been established.

### 5.3 Safety monitoring and drug interaction management

Safety monitoring and interaction assessment should be prespecified in prospective HUAED studies. Febuxostat was associated with a higher incidence of ED than allopurinol in one observational cohort (8), whereas febuxostat, benzbromarone, and compound 3170 improved erectile-function-related outcomes in hyperuricemic animal models (1, 36). These discordant findings require prospective evaluation and should not be translated into categorical prescribing recommendations. Benzbromarone also warrants attention to regional availability, hepatic risk, and liver-function monitoring. In specific HUA studies, Yin Chen Wu Ling San showed a short-term urate-lowering signal (55), and a real-world analysis reported gouty-arthritis and liver-injury outcomes for CHM relative to benzbromarone (56); neither establishes safety or efficacy for all CHM products or for HUAED. Because herbal formulas contain multiple constituents, protocol-specific pharmacokinetic and pharmacodynamic interactions should be assessed (61). Monitoring may include liver and kidney function, electrolytes, cardiovascular status, sexual function, hormones when indicated, and product-specific interaction testing. Acupuncture and other external therapies also require standardized procedures and adverse-event reporting (62, 63).

**Figure 3** presents a hypothesis-generating research framework rather than a clinical decision tree or validated treatment algorithm. The proposed research populations are characterized by metabolic-inflammatory, hypogonadal/neurovascular, or hyperuricemic features; these research phenotypes are not mutually exclusive clinical categories or established treatment strata. Guideline-based management is presented as background care, whereas integrative approaches are designated only as candidate interventions requiring controlled evaluation of efficacy and safety. The proposed outcomes requiring validation include metabolic and erectile outcomes, hormonal and erectile outcomes, and serum urate, vascular, and safety outcomes. Urate-lowering therapy (ULT) should be used only when independently indicated according to applicable hyperuricemia or gout guidelines and not on the basis of age, erectile dysfunction status, or research-phenotype assignment alone. No HUAED-specific urate threshold, validated phenotype-specific treatment pathway, or established efficacy of an integrative intervention is implied.

**Figure 3:**
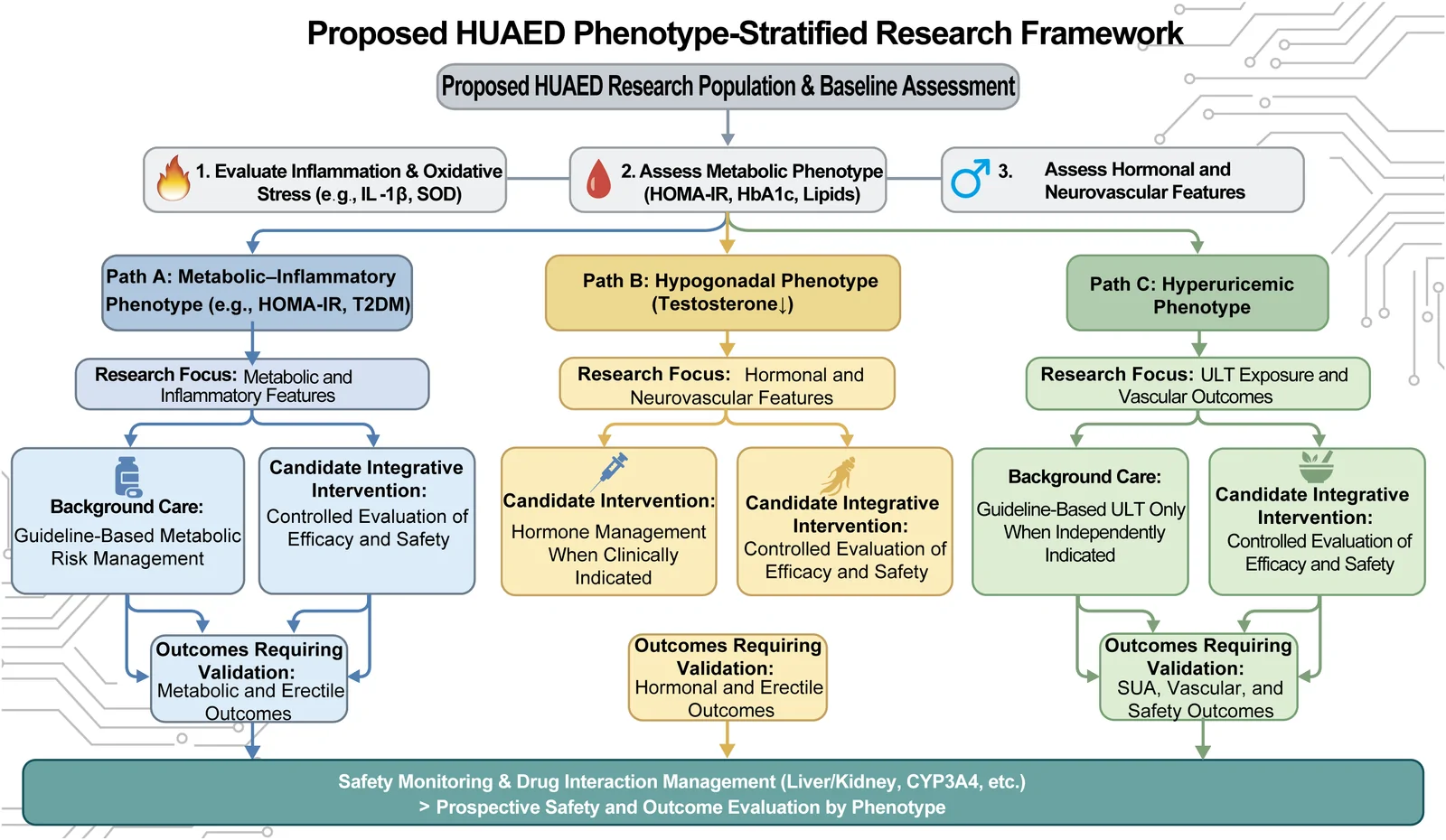
Proposed phenotype-stratified research framework for HUAED.

### 5.4 Illustrative operational validation strategy

For prospective validation, HUAED may be operationally defined as ED symptoms persisting for at least three months with IIEF-5 ≤21, together with fasting SUA >420 μmol/L on two separate measurements, after documenting temporal coexistence and assessing alternative causes of ED. Gout and asymptomatic HUA should be prespecified as separate clinical strata. Diabetes may be included if treatment and glycemic status are stable and should be stratified or adjusted; suspected hypogonadism should require two morning testosterone measurements and be managed according to endocrine guidance. Previous ULT, PDE5 inhibitor, testosterone, and herbal exposure should be recorded, with protocol-defined washout when ethical or stable background treatment when clinically indicated. Metabolic-inflammatory, hormonal, and neurovascular domains may overlap within the same participant.

An illustrative add-on trial would enroll adults meeting this operational definition after stabilization of guideline-based HUA/gout, ED, and cardiovascular-risk care. Participants would be randomized, double-blind, to a chemically and batch-standardized herbal product or matched placebo for 12 weeks while indicated background ULT and/or PDE5 inhibitor therapy remains unchanged. The primary endpoint would be between-group change in IIEF-EF at week 12. Secondary mechanistic endpoints would include SUA, HOMA-IR, HbA1c, lipids, inflammatory/oxidative markers, morning testosterone when indicated, penile Doppler measures, and exploratory MLCK/MLC2-related biomarkers; safety endpoints would include adverse events, liver/kidney function, and protocol-specific interaction testing, with durability assessed to week 24. Sample size should be based on the prespecified minimal clinically important difference in IIEF-EF, variance from pilot data, two-sided α=0.05, at least 80%–90% power, and expected attrition. This two-arm comparison tests incremental benefit, not synergy; synergy would require a 2×2 factorial design containing each component alone, the combination, and a common control, with a prespecified interaction term.

## 6 Future perspective

Future HUAED research should prioritize disease-specific validation rather than extrapolation. First, studies should quantify both neural initiation and endothelial maintenance of erection, including cavernous-nerve function, nNOS/eNOS signaling, sGC redox state, and cGMP generation. Second, the balance between MLCK-mediated MLC2 phosphorylation and RhoA/ROCK-MYPT1-mediated MLCP inhibition should be studied across disease stages (1, 49). Third, Nrf2 represents a mechanistically important research gap. High urate suppressed Nrf2-related defense in endothelial cells (39), whereas Nrf2 activation improved diabetic and hypertension-associated ED in animal models (40, 41); suitable urate concentrations were protective in an ox-LDL endothelial model (42). Direct studies should therefore define urate concentration, compartment, exposure duration, Nrf2/HO-1 activity, and NLRP3 coupling in HUAED penile tissue. Fourth, candidate CHM-derived interventions such as Paeonia veitchii, Pueraria formulas, and punicalagin should be evaluated with standardized chemistry and disease-specific endpoints (54, 57, 64). Fifth, microbiome research should test the peroxisome proliferator-activated receptor gamma (PPARγ)-ATP-binding cassette subfamily G member 2 (ABCG2) urate-excretion pathway and gut-kidney mechanisms in HUAED rather than assume transferability from HUA models (65, 66). Stem-cell-derived extracellular vesicles are emerging candidates for ED repair (67, 68), but no direct evidence currently supports their use for HUA-induced structural injury. Wearable urate sensing (69), single-cell atlases of human corpus cavernosum (70), and multi-omics approaches (71) may improve phenotyping and biomarker discovery. A real-world HUA study comparing CHM with benzbromarone (56) should not be interpreted as evidence of synergistic HUAED treatment. Ultimately, prospective trials must determine whether integrated interventions improve erectile function, endothelial measures, quality of life, and safety beyond guideline-based care (72).

For herbal formulations and candidate compounds, future work should follow a staged causal-validation pipeline. First, orthogonal chemical profiling should define batch composition and quantify parent compounds, metabolites, and circulating/exposure-relevant components. Second, unbiased transcriptomic, proteomic, metabolomic, or single-cell screening may nominate pathways, but network-pharmacology predictions and expression changes should be treated only as hypothesis-generating. Third, direct target engagement should be demonstrated with complementary biophysical or cellular assays, such as surface plasmon resonance, cellular thermal shift assays, affinity pull-down, or validated occupancy probes. Fourth, genetic perturbation (knockdown, knockout, or target-mutant constructs) and selective pharmacological perturbation should test necessity and specificity. Finally, rescue experiments should restore the proposed target or downstream function and link engagement to erectile, vascular, inflammatory, or contractile phenotypes in HUAED models. The Wumei Pill study combining HPLC-based characterization of circulating components with proteomics, network pharmacology, and experimental validation provides an example of linking formulation chemistry to a candidate STAT3-related mechanism (73). The solasonine study illustrates a stronger compound-to-target workflow using cellular thermal shift and surface plasmon resonance assays, target perturbation, and functional rescue to evaluate APT2 binding, STAT3 depalmitoylation, and ferroptosis (74). These oncology studies are methodological examples only and do not constitute efficacy or mechanistic evidence for HUAED.

## 7 Limitations

This narrative, hypothesis-generating review did not employ a PRISMA flow diagram, formal risk-of-bias assessment, or certainty-of-evidence grading. Although the structured search is fully reported in **Supplementary Table S1** and **Supplementary Appendix S1**, only 23 core records were retained from database screening, with additional studies identified through citation tracking and author knowledge. This supplementation was necessary because direct HUAED evidence is sparse, but it may introduce selection and confirmation bias. No language restriction was applied and one Chinese-language guideline was included; however, CNKI, Wanfang, SinoMed, and other Chinese-language databases were not searched, so Chinese TCM studies may be under-represented. The marked PubMed–Web of Science yield difference also reflects non-equivalent indexing and field mapping. The evidence base is heterogeneous and relies heavily on animal, cellular, HUA-only, or ED-only studies. Several TCM mechanistic studies originated from the same group and lack independent replication. Consequently, the proposed causal links, TCM-biomedical mappings, phenotype strata, and integrated interventions should be interpreted as research hypotheses rather than validated clinical recommendations.

## 8 Conclusion

HUAED is a clinically relevant but incompletely validated construct linking abnormal urate metabolism with male sexual dysfunction. Current evidence supports an association in some populations and several biologically plausible mechanisms, but does not establish a uniform causal relationship, an ED-specific urate threshold, or the clinical effectiveness of integrated Chinese and Western medicine for HUAED. The prevention-regulation-repair framework proposed here therefore serves as a structured research model. Its neurovascular, Nrf2, inflammatory, contractile, metabolic, hormonal, and structural components require independent mechanistic confirmation and prospective clinical testing before they can inform routine care.

## Supplementary material

Supplementary Table 1 PubMed and Web of Science search strategy and study screening process.

Supplementary Table 2 Citation–evidence cross-reference and reference verification.

Supplementary Appendix S1 Search-term development and keyword framework.
