# Supplementary material for: Hyperuricemia-related erectile dysfunction: mechanistic links and a translational framework for integrated traditional Chinese and western medicine
Source: Front Endocrinol (Lausanne). 2026 Oct 22;17:1911364. doi: 10.3389/fendo.2026.1911364 (PMC13736097; doi:10.3389/fendo.2026.1911364)
Supplement: Supplementary file 1 [file SupplementaryFile1.docx]

**Supplementary Appendix S1**

Search-Term Development and Database-Specific Keyword Framework

# Purpose and logic

This appendix documents the exact concept blocks used for the structured component of a narrative, hypothesis-generating review. Because the review synthesizes HUA-only, ED-only, and directly overlapping HUAED evidence, the condition block combines hyperuricemia/uric-acid terms with erectile-dysfunction terms using OR. The therapeutic block is then combined using AND. This broad strategy was intentionally supplemented by citation tracking and author knowledge because direct HUAED studies were sparse.

# PubMed strategy

("Hyperuricemia"[Mesh] OR hyperuricemia[Title/Abstract] OR "uric acid"[Title/Abstract] OR "Erectile Dysfunction"[Mesh] OR "erectile dysfunction"[Title/Abstract] OR ED[Title/Abstract]) AND ("Medicine, Chinese Traditional"[Mesh] OR "Drugs, Chinese Herbal"[Mesh] OR Acupuncture[Mesh] OR "Integrative Medicine"[Mesh] OR "traditional Chinese medicine" OR TCM OR "integrated Chinese and Western medicine" OR "Western medicine" OR "conventional treatment")

# Web of Science Core Collection strategy

TS=(hyperuricemia OR "uric acid" OR "erectile dysfunction" OR ED) AND TS=("traditional Chinese medicine" OR TCM OR "Chinese herbal medicine" OR acupuncture OR "integrative medicine" OR "integrated Chinese and Western medicine" OR "Western medicine" OR "conventional treatment")

**Database adaptation note:** PubMed field tags and MeSH headings were translated into the Web of Science Topic field (TS). No language restriction was applied. The search cutoff was August 10, 2026.

**Explanation of database-yield differences**

The PubMed and Web of Science record counts should not be interpreted as directly comparable measures of database coverage. PubMed combined MeSH terms with Title/Abstract terms, while untagged intervention terms were processed using PubMed-specific automatic term mapping. Web of Science used the Topic field, which searches titles, abstracts, author keywords, and Keywords Plus. In addition, the PubMed strategy was intentionally sensitive because the review synthesized HUA-only, ED-only, and directly overlapping HUAED evidence. The standalone abbreviation “ED” and the broad intervention terms generated non-specific records, including records in which “ED” referred to concepts other than erectile dysfunction; these records were removed during title/abstract screening. Of the six records retrieved from Web of Science, five overlapped with PubMed and one remained unique after cross-database deduplication. Therefore, the marked difference in initial yield primarily reflects database-specific indexing and query processing together with the intentionally broad PubMed strategy, rather than a difference in the eligibility criteria applied during screening.

**Screening and resolution of disagreements**

Initial title/abstract screening was performed by SH. All 23 core records retained from the structured search were independently verified against the eligibility criteria by YNF. Disagreements were resolved through discussion and consensus; any unresolved disagreement was adjudicated by YFZ. Because category-specific exclusion reasons were not prospectively preserved for all excluded records, retrospective exclusion counts were not fabricated.

**Selection of additional references**

Additional references were identified through backward citation tracking, forward citation tracking, and the authors’ prior knowledge of the field. These records were retained only when they addressed a prespecified epidemiological, mechanistic, clinical, safety, or methodological domain; provided verifiable peer-reviewed evidence or an authoritative clinical guideline; supported a clearly identifiable statement in the manuscript; were available before the search cutoff; and did not duplicate a structured-search record. Additional references were evaluated using the same relevance and evidence-directness criteria as database-derived records. Their individual identification routes are reported in Supplementary Table S2. These supplementary references were not counted among the 23 core records retained from the structured database search.

**Chinese-database coverage**

No dedicated Chinese bibliographic database was searched because Chinese-language databases (e.g., CNKI, Wanfang, and SinoMed) were not searched because the predefined structured component was limited to PubMed and Web of Science and the review was not intended to be exhaustive. However, no language restriction was applied to the PubMed and Web of Science searches, and Chinese-language records indexed in these databases were eligible; one Chinese-language hyperuricemia/gout practice guideline was retained. We acknowledge that the absence of a dedicated search of SinoMed, CNKI, Wanfang, or VIP may have resulted in the omission of locally indexed TCM studies. This limitation is stated explicitly in the revised manuscript, and evidence derived from internationally indexed TCM studies is not presented as a comprehensive representation of the Chinese-language literature.

**Structured evidence appraisal**

For studies supporting the principal conclusions, we systematically recorded the study design, population or experimental model, sample size when available, comparator, principal outcome or effect estimate, directness to HUAED, and key methodological limitations. Evidence was classified as direct human HUAED evidence, direct preclinical HUAED evidence, indirect HUA-only evidence, indirect ED-only evidence, or contextual/methodological evidence. Because the included evidence ranged from clinical guidelines and meta-analyses to observational, animal, cellular, and narrative studies, no single pooled quality score was calculated. The design, directness, limitations, and role of each study in the synthesis are reported in Supplementary Table S2.

# Final screening summary

| Measure | Final verified value |
| --- | --- |
| PubMed / Web of Science | 2,447 / 6 records |
| Total identified | 2,453 records |
| Duplicates removed | 6 records |
| Unique records screened | 2,447 records |
| Excluded at title/abstract screening | 2,424 records |
| Core records retained | 23 records |
| Language restriction | None; one Chinese-language practice guideline was eligible |
| Additional references identified through citation tracking or author knowledge | 51 records |
| Final citation–evidence audit | 74 references |

# Eligibility and narrative supplementation

Records were excluded when they failed one or more eligibility criteria: no direct HUA-ED focus; an unrelated disease or outcome; no relevant mechanistic or clinical link; or no relevance to the conventional, TCM, or integrative intervention domains. Category-specific exclusion counts were not retrospectively fabricated because they were not preserved in the archived screening export.

Additional studies were identified through backward/forward citation tracking and the authors' knowledge of the field. This supplementation can introduce selection and confirmation bias; the risk is stated explicitly in Section 7 (Limitations) of the revised manuscript. The complete database exports and the 23 retained core records are provided in Supplementary Table S1, and the final 74-reference citation-evidence audit is provided in Supplementary Table S2.
